# Extensor tendon traumatic gap reconstruction

**DOI:** 10.1186/1753-6561-9-S3-A69

**Published:** 2015-05-19

**Authors:** Tolga Turker

**Affiliations:** 1 Department of Surgery, University of Arizona, Tucson, Arizona 85721, USA

## 

Repair of extensor tendon defects or gap injuries often require complicated surgical techniques. Unlike flexor tendons, which have good excursion that can help mobilize the flexor tendon allowing for repair of up to 1 cm gaps, extensor tendons only have 1-2 mm of excursion, especially in Verdan’s extensor Zones 1 to 5. Furthermore, a 1-mm tendon gap in Zones 1 to 5 may cause 20 degrees extension loss or shortening of the extensor tendon, and a gap even as small as 1 mm may cause decreased finger flexion. Due to such significant potential damage and loss of function caused by extensor tendon defect injuries, especially in Zones 1-5, solutions most often require some type of reconstruction techniques.

## The zone 1

According to the size of defects three major reconstruction techniques may be used. *Terminal tendon hemilateral band technique;* This technique uses the lateral bands of the same injured fingers to bridge the gap formation. This technique requires temporary fixation distal interphalaheal joint (DIPJ) with a K wire for 4 weeks. After the wire removal, an aluminum splint is used for following 2 more weeks with occupational therapy (OT).

*Interpostional tendon grafting;* This technique requires a free tendon harvesting in order to reconstruct the extensor tendon defects. The Palmaris longus tendon is sutured to the distal phalanx then the tendon sutured to the proximal part of the extensor tendon that is being reconstructed and the DIPJ is immobilized in 0-15 degrees hyperextension for 4weeks then OT is started. If there is soft tissue defect with tendon loss, composite tissue, using palmarislongus and skin island as Inoue et al. reported. Wavreille et al. demonstrated in a cadaveric study that uses second toe extensor apparatus with skin island that’s blood is supplied by the first dorsal metatarsal artery.

*Tendon transplantation and allograft;* If there is an injury that involves more then one finger, one finger has tendon defect in zone 1 or 2 with good condition of DIPJ and PIPJ and the other finger or fingers have severe injury that requires either DIPJ either needs fusion or amputation, this finger(s)'s terminal tendon can be harvested to reconstruct the defect in extensor tendon. It is also possible to use allograft as a tendon resource.

## The zone 2

Zone 2 defects show similarities to the zone 1 defects. Again local tendon grafts and free interpositional tendon reconstructions may be considered.

* Local tendon grafts;* Kochevar et al.'s cadaveric study uses 0.5 cm defect, which can be bridge with preparation of tendon graft from proximal portion and flip over to reconstruct the gap.

*Interpositional tendon grafts;*Similar to the zone 1 extensor tendon defects, PL tendon may be used to reconstruct the zone 2 defects.

## The zone 3

This zones defects are important because they may cause boutonnière deformity. Therefore reconstruction is important not only overcome the defect but also prevent the boutonnière deformity.

If lateral bands and/or proximal portion of the tendon are intact, one of two techniques can be used. First, the lateral bands can be divided longitudinally and centered over the PIP joint and sutured to each other. Second, a distal based tendon flap can be created using the proximal of tendon and flip over to distally to overcome the defect as Kochevar described in their cadaveric study.

Again, composite tissue, Palmaris longus and venous island flaps can be used to reconstruct the extensor tendon defects in zone 3.

## The zones 4 and 5

In this zone, there is more tendon substance then other zones, therefore reconstruction of tendon gaps could be relatively easier. Again, two major reconstructions techniques should be in mind. Local tendon flaps and interpositional free tendon reconstructions.

like in zone 2, Kochevar et al. reported local tendon graft technique that can bridge 1 cm tendon defects without repair site suture failure with maximum IP and MP joint flexion.

Interpostional tendon reconstructions also another solution to overcome tendon defects in zone 4 defects. PL tendon is again, frequently used as a tendon graft in those defects. If there is a skin defect, composite venous flaps and PL tendon reconstruction could be an alternative procedure to fix gap formation in this zone.

## The zones 6, 7 and 8

If adjacent tendons are in continuity, the distal end of the defective tendon maybe sutured to neighbor tendon side to side fashion in order to get the intact tendons motor power.

Interpostional tendon grafting: PL tendon can be used to reconstruct the extensor tendon in these zones. The tendons are generally sutured using the Pulvertaft technique which allows early rehabilitation. If there is any skin defects, as Scheker et al reported, tendon reconstruction may be combined with either lateral arm or groin flaps for coverage.
